# Supplementary material for: The Rice Pentatricopeptide Repeat Protein PPR756 Is Involved in Pollen Development by Affecting Multiple RNA Editing in Mitochondria
Source: Front Plant Sci. 2020 Jun 12;11:749. doi: 10.3389/fpls.2020.00749 (PMC7303307; doi:10.3389/fpls.2020.00749)
Supplement: Supplementary file 1 [file Data_Sheet_1.PDF]

SUPPLEMENTARY DATA

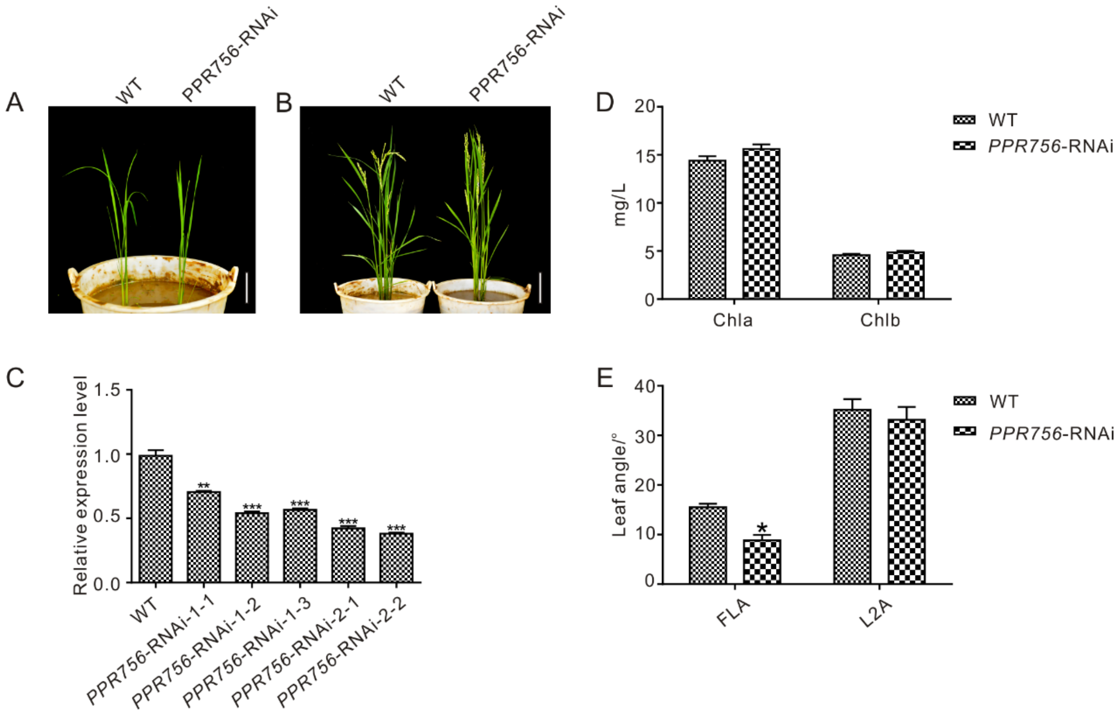

Supplementary Figure 1. Phenotypic characterization of *PPR756* RNAi lines.

(A, B) Plant phenotype comparison of the WT and *PPR756* RNAi lines at rice seedling and reproductive stage. Bars 5 cm for **(A)** and 10 cm for **(B)** respectively. **(C)** The relative expression level of *PPR756* transcript in *PPR756* RNAi lines were detected by real-time qPCR. **(D)** The chlorophyll a (Chla) and chlorophyll b (Chlb) content of leaves in the WT and series of independent *PPR756* RNAi lines. The unit of content is mg/L. **(E)** Comparison of leaf angles in the WT and *PPR756* RNAi lines. FLA, flag leaf angle, L2A, the last second leaf angle. The unit is "o". All quantitative data were means ± SD based on three independent experiments (Student's t-test; *, $P < 0.05$; **, $P < 0.01$; ***, $P < 0.001$).

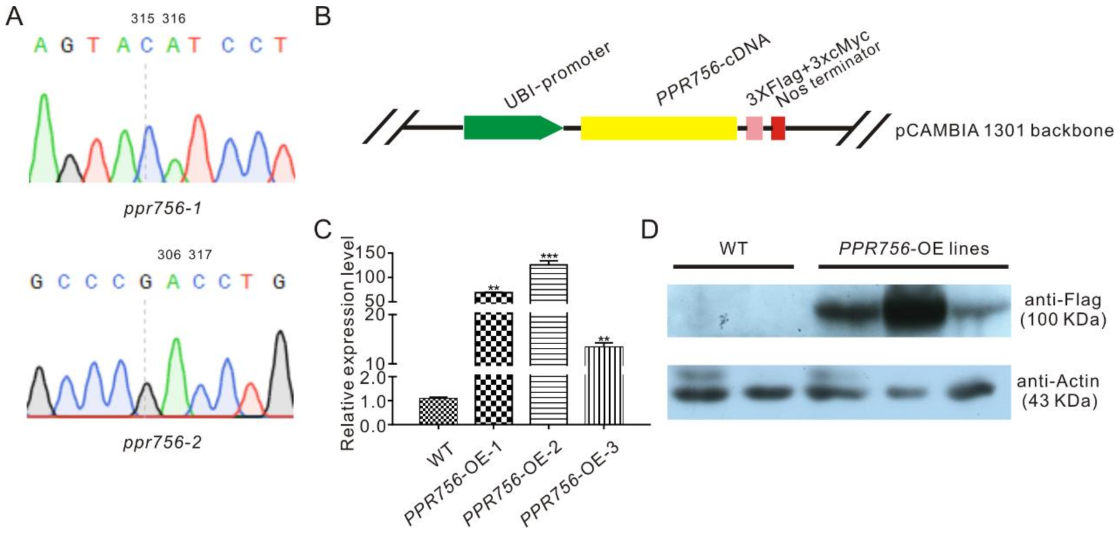

Supplementary Figure 2. Construction and validation of *PPR756* transgenic lines. **(A)** The sequencing analysis of *ppr756* mutants. **(B)** The structure scheme of *PPR756* overexpression construct. **(C)** The transcript level detection in the WT, three independent *PPR756* OE lines. Rice *actin* gene served as a control. **(D)** Immunoblot assays in the WT three independent *PPR756* OE lines. Actin was served as a control, and Flag signal represents the fusion protein PPR756-Flag-cMycs. Quantitative data were means ± SD based on three independent experiments (Student's t-test; *, $P < 0.05$; **, $P < 0.01$; ***, $P < 0.001$).

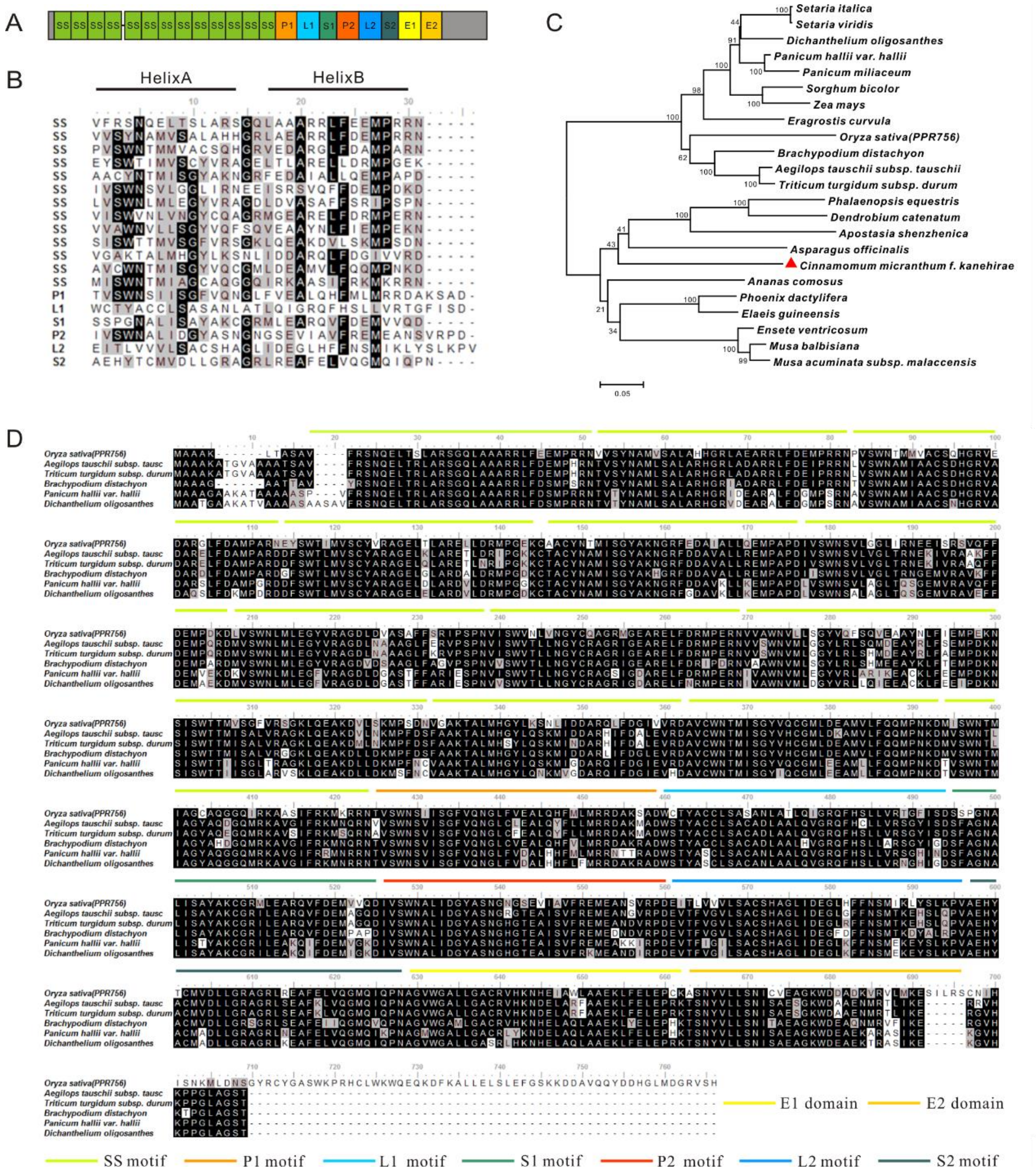

Supplementary Figure 3. Structure and sequence alignment of PPR756 protein.

(A) PPR and E motifs of PPR756 protein are indicated and marked respectively. **(B)** Amino acids sequence alignment of 19 PPR motifs of PPR756, highly conserved amino acids are marked in dark. **(C)** Phylogenetic tree analysis of PPR756 homologs. The analysis was constructed by MEGA 5.2 program and showed the homologs over 55% similarity including including *Aegilops tauschii*, *Triticum turgidum*, *Brachypodium distachyon*, *Panicum hallii*, *Dichanthelium oligosanthes*, *Setaria italic*, *Eragrostis curvula*, *Setaria viridis*, *Sorghum bicolor*, *Zea mays*, *Panicum miliaceum*, *Phoenix dactylifera*, *Ananas comosus*, *Ensete ventricosum*, *Cinnamomum micranthum f. kanehirae*, *Musa balbisiana*, *Asparagus officinalis*, *Musa acuminata subsp.*

Malaccensis, Phalaenopsis equestris, Apostasia shenzhenica, Dendrobium catenatum were almost monocots, and only one was a dicot (marked by triangle). **(D)** Detailed sequence alignment of rice PPR756 protein with some orthologs. The alignment showed very highly conservation among PPR756 and its orthologs. The different motifs are marked using various colors of straight lines.

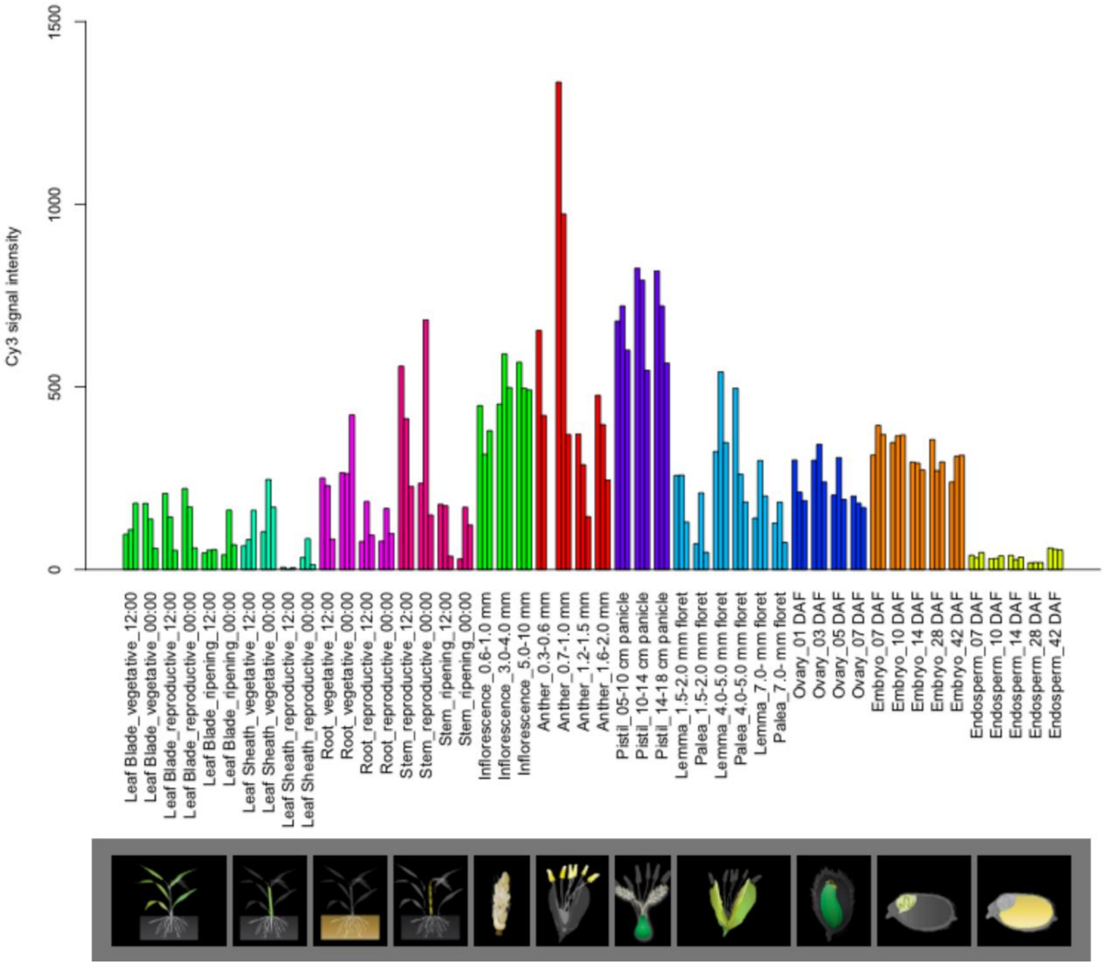

Supplementary Figure 4. Predicted spatio-temporal expression pattern of PPR756.

Predicted spatio-temporal expression pattern of PPR756 from the RiceXPro database.

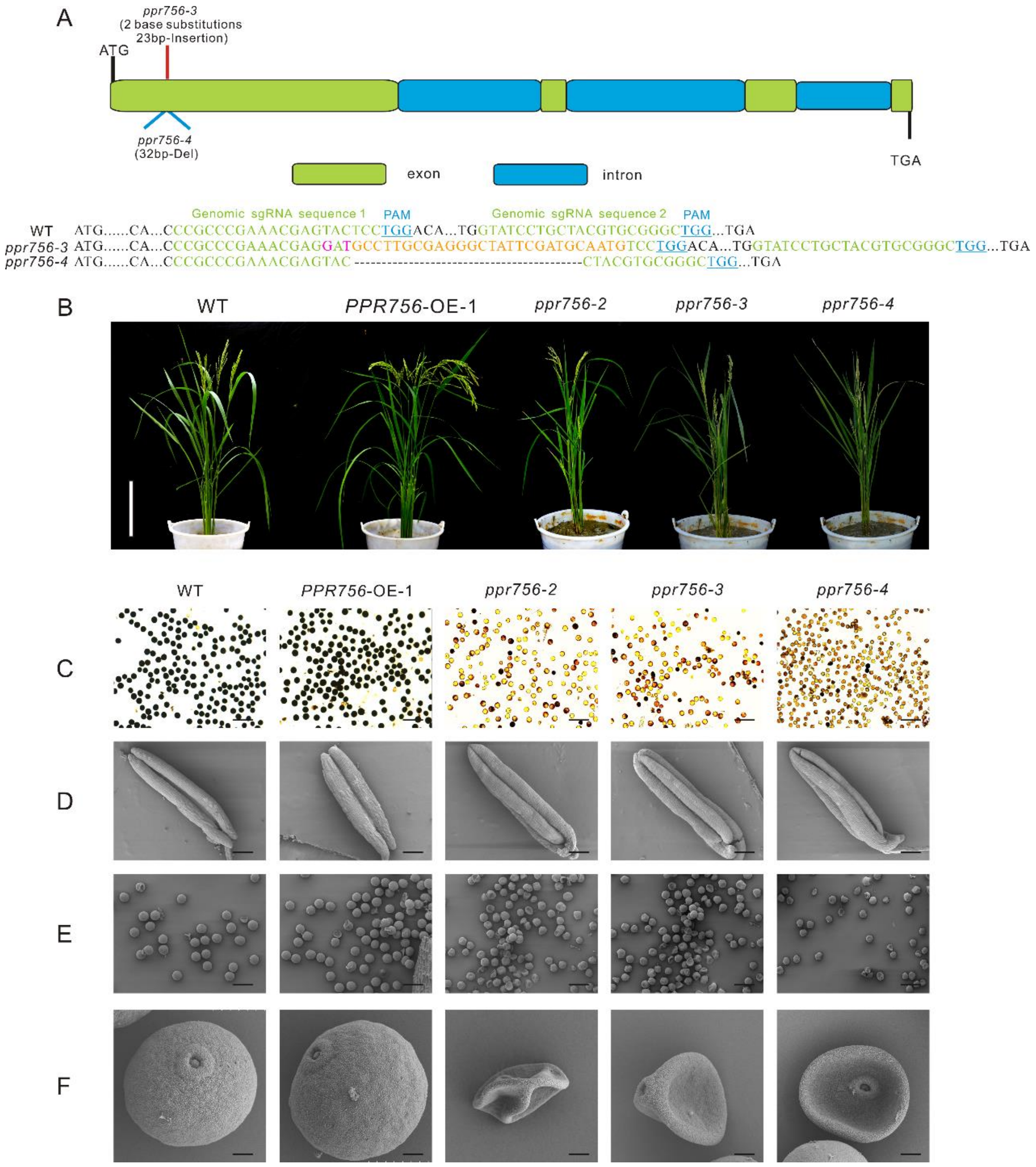

Supplementary Figure 5. Phenotypic characterization, fertility detection and scanning electron microscopy observations in the WT, *PPR756* OE line and *ppr756* mutant lines. **(A)** The structures of *PPR756* and *ppr756* mutants. Other *ppr756* mutant lines showed with 2 base substitutions 23 bp insertion in *ppr756-3* line and 32 bp deletion in *ppr756-4* line. **(B)** Plant phenotypes comparison of the WT, PPR756 OE line and *ppr756-2*, *ppr756-3*, *ppr756-4* mutant lines at reproductive stage. Bars, 20 cm. **(C)** Comparison of pollen fertility of WT, the *PPR756* OE line, and *ppr756* mutant lines by potassium iodide (1% I_2-KI) staining. The darkly stained pollen is fertile, whereas the lightly or non-stained pollen is sterile. Bars, 200 μm. **(D)** Scanning electron microscopy (SEM)

observation of anthers of WT, the *PPR756* OE line, and *ppr756* mutant lines. Bars, 200 μm. Representative results from three independent experiments. **(E, F)** Scanning electron microscopy (SEM) observation of pollens of the *PPR756* OE line and *ppr756* mutant lines. Bars, 100 μm for **(E)** and 10 μm for **(F)**.

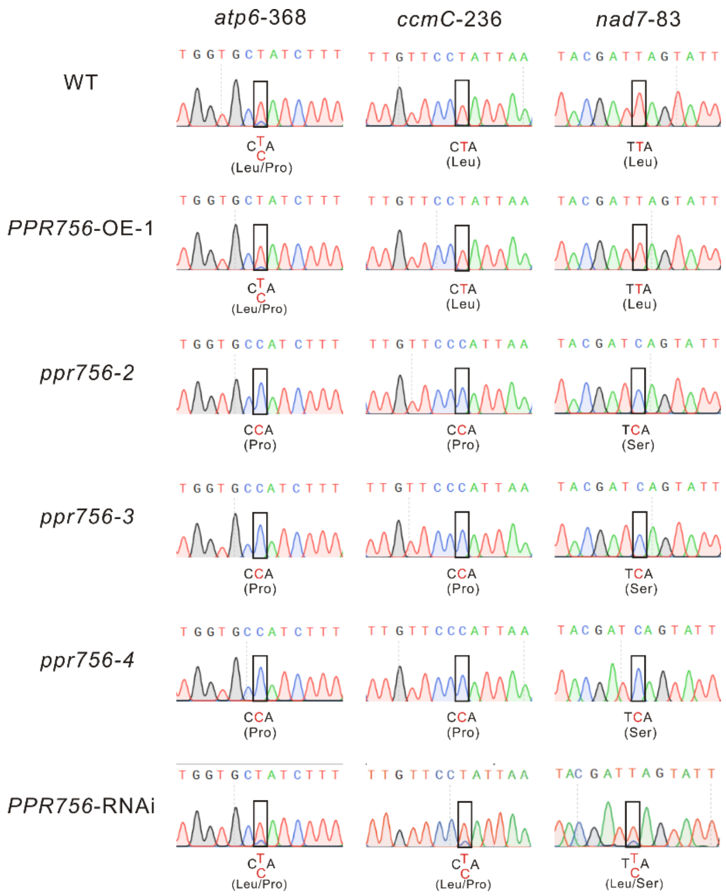

Supplementary Figure 6. RNA editing analysis in the WT, *PPR756* OE line, *ppr756* mutant lines and *PPR756* RNAi line.

RNA editing analysis of *atp6-368, ccmC-236, nad7-83* in the WT, *PPR756* OE line, *ppr756-2*, *ppr756-3*, *ppr756-4* mutant lines and *PPR756* RNAi line.

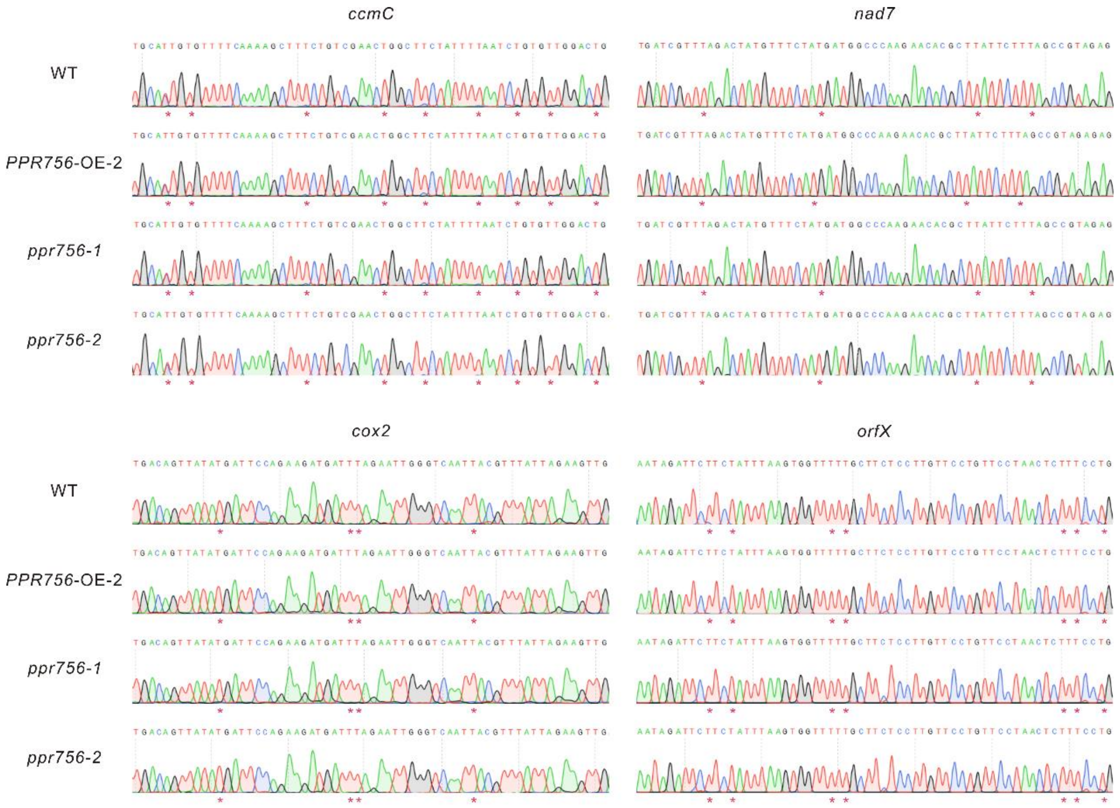

Supplementary Figure 7. RNA editing analysis of unaffected sites in the WT, *PPR756* OE line and *ppr756* mutant lines.

RNA editing analysis of unaffected mitochondrial edited sites in the WT, *PPR756* OE line and *ppr756* mutant lines. The edited sites are marked by “*”.

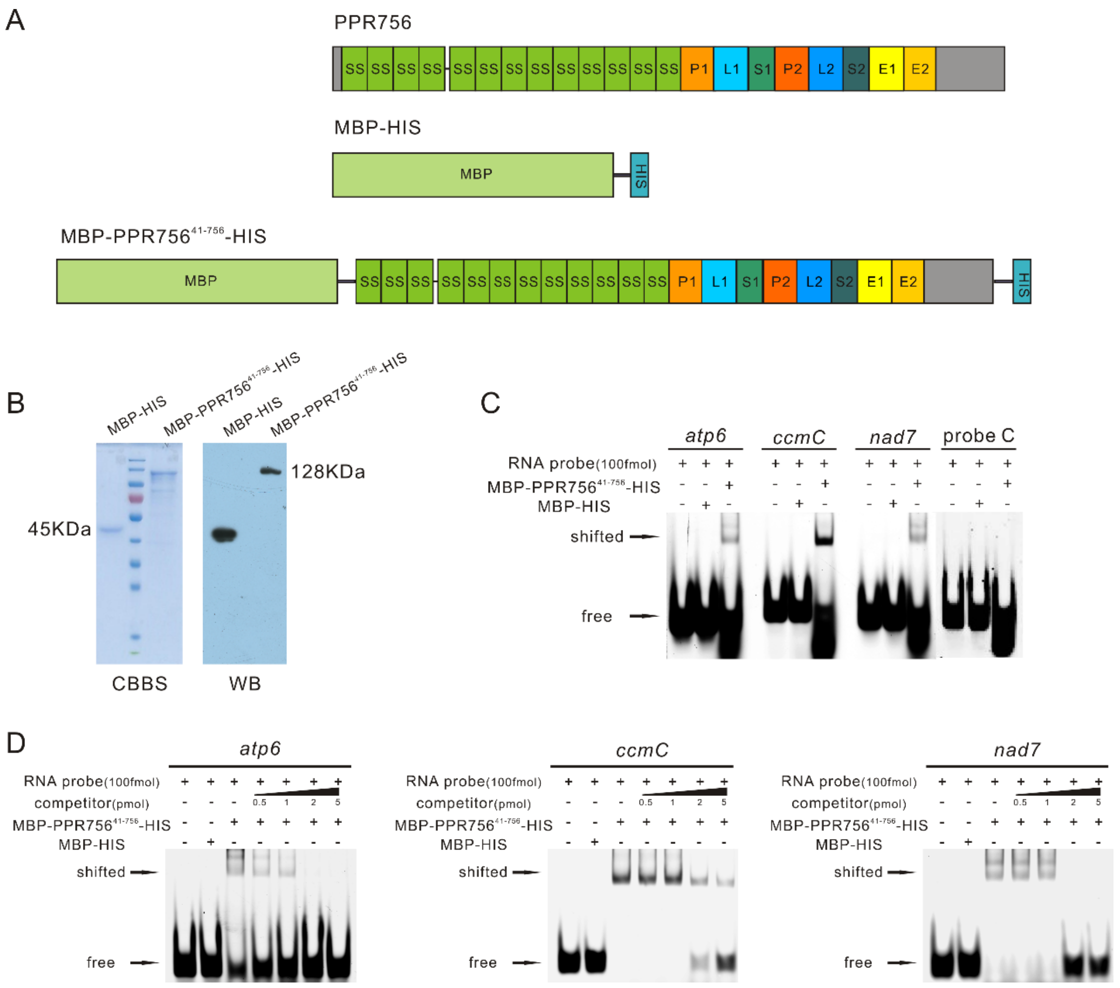

Supplementary Figure 8. PPR756 can directly bind to its target genes in vitro.

(A) Schematic structures of expressed recombinant proteins. **(B)** The expressed and purified recombinant proteins were validated by Coomassie brilliant blue staining (left) and Western blots (right). CBBS, Coomassie brilliant blue, WB, Western blot. **(C)** RNA electrophoresis mobility shift assays (REMSAs) of the recombinant MBP-PPR756$^{41-756}$-HIS protein and MBP-HIS tags with the FAM-labeled RNA probes. MBP-HIS tags and probe C served as a negative control. **(D)** The corresponding non-labeled RNA probes in a concentration gradient were used as competitors for the REMSA of MBP-PPR756$^{41-756}$-HIS protein with its target probes. MBP-HIS tags served as a negative control.

Table S1 Primers used in this study

Name	Sequences(5'-3')	Purpose
PPR756-RNAi-F	GGGGACAAGTTTGTACAAAAAAGCAGGCTGGAAGCTCACCGCCTCTGCTGTGTTTA	RNAi
PPR756-RNAi -R	GGGGACCACTTTGTACAAGAAAGCTGGGTC ACCAGCCCGCACGTAGCAGGAT	
PPR756-cDNA-F	ATGGCAGCTGCGAAGCTCAC	Amplification
PPR756-cDNA-R	ATGACTTACTCTGCCATCCATGAGC	
PPR756-RT-F	AGCGTAATGTTGTGGCTTGGAATGT	RT-PCR
PPR756-RT-R	GGAATACTGCTATCACCTCAGACCC	
PPR756-HBT-F	CTTGCTCCGTGGATCCGCCACCATGGCAGCTGCGAAGCTCAC	Subcellular localization
PPR756-HBT-R	TGCTCACCATGGATCCATGACTTACTCTGCCATCCATGAGC	
PPR756-qPCR-F	GCCTTGATTTCTGCCTAT	qRT-PCR
PPR756-qPCR-R	ACCTCAGACCCATTACCA	
PPR756$^{41-756}$-Pmal-2X-F	ATCCTCTAGAGTCGACAACGTGGTCTCCTACAACGCGAT	Prokaryotic expression
PPR756$^{41-756}$-Pmal-2X-R	TTGCCTGCAGGTCGACGCAGCCGATCTCAGTGGT	
PPR756-*BIFC-F*	CGCCACTAGTGGATCCGCCACCATGGCAGCTGCGAAGCTCAC	BiFC
PPR756-BIFC-R	TACTATCGATGGATCCATGACTTACTCTGCCATCCATGAGC	
PPR756-pro-F	GGAATTCCTCTATCCAAAACTGACAAACCAAGG	GUS expression
PPR756-pro-R	GGAATTCCTGCCGCCAGATACCACAA	
PPR756-OE-F	CGACTCTAGAGGATCGCCACCATGGCAGCTGCGAAGCTCAC	Overexpression
PPR756-OE-R	GTTTCTGCTCGGATCGCCACCATGACTTACTCTGCCATCCATGAGC	
PPR756-Y2H-F	GGCCATGGAGGCCGCCACCATGGCAGCTGCGAAGCTCAC	Yeast two-hybrid assays

PPR756-Y2H-R	GGCCTCCATGGCCATATGACTTACTCTGCCATCCATGAGC	
actin-Q-F	CTTCATAGGAATGGAAGCTGCGGGTA	qRT-PCR
actin-Q-R	CGACCACCTTGATCTTCATGCTGCTA	
atp6-qpcr-F	ATTTGGTCTTGATATGGGTA	
atp6-qpcr-R	ATTTGGCACTGACTTTCC	
ccmC-qpcr-F	GAACAAATAGGTGGAAATG	
ccmC-qpcr-R	GAGCCAAAGTAATGAGAA	
nad7-qpcr-F	ACGGAGAAGTGGTGGAAC	
nad7-qpcr-R	GCTGAAGAATGAGCGTGT	
nad5-qpcr-F	GACAAGGGTGCTATTGAG	
nad5-qpcr-R	GAGTCCCACATACGAGAA	
GAPDH-QPCR-F	AAGCCAGCATCCTATGATCAGATT	
GAPDH-QPCR-R	CGTAACCCAGAATACCCTTGAGTTT	
PPR756-crispr-U6a-F	GCCGGTATCCTGCTACGTGCGGGC	CRISPR sytem
PPR756-crispr-U6a-R	AAACGCCCGCACGTAGCAGGATAC	
PPR756-crispr-U3-F	GGCACGCCCGAAACGAGTACTCC	
PPR756-crispr-U3-R	AAACGGAGTACTCGTTTCGGGCG	
PPR756-gRT-U6a+	GTATCCTGCTACGTGCGGGCGTTTTAGAGCTAGAAAT	
PPR756-gRT-U3+	CGCCCGAAACGAGTACTCCGTTTTAGAGCTAGAAAT	
PPR756-OsU6aT-	GCCCGCACGTAGCAGGATACGGCAGCCAAGCCAGCA	
PPR756-OsU3T-	GGAGTACTCGTTTCGGGCGTGCCACGGATCATCTGC	
CAS9-PCR-F	CCACTCCATCAAGAAGAACCTCATC	
CAS9-PCR-R	CGATAAGGAAGTGACCACGGAAC	
PPR756-TEST-F	GGTCTCCTACAACGCGATGGTCT	
PPR756-TEST-R	CACAACATTACGCTCAGGCATTCTA	
atp6-FAM	UAUGGCGGUAACUGUCGUUUUGGUGCCAUCUUUAU	RNA-EMSA

ccmC-FAM	ACAGCUAUAAACAGUUUCUUGUUCCCAUUAACAA	
nad7-FAM	UCCUGCUGCUCAUGGUGUUUUACGAUCAGUAUUCC	
nad5-FAM	AAAACUAAUACCUAUUCUGUUUAGUACUUCAGGUG	
atp1-1-F	ATGGAATTCTCACCCAGAGC	Mitochondrial RNA editing assay
atp1-1-R	TATAGGAACCAGGCTATCCA	
atp1-2-F	TAGAAAGAGCCGCTAAAC	
atp1-2-R	CTAATTAATCTCCTTCGCAG	
atp6-1-F	AATTACTCATTTTAATGGAG	
atp6-1-R	CCACTGCCATTAGCACCTTT	
atp6-2-F	GAAAATGACTTGTCACTGTG	
atp6-2-R	TTTCGATTACAATCATGTGG	
atp9-F	GCAAAGTCAAGTCTCCACGA	
atp9-R	CAAAGAGAGATATCTACACC	
ccmB-F	ATGAGACGACTCTTTCTTGA	
ccmB-R	TCAATCTTGTAAACTAATCG	
ccmC-F	ATGTCAGTTTCGTTATTACA	
ccmC-R	CTAGGTTTTTAGTGGTATTC	
ccmFc-1-F	GGTCCAACTACAGAACTTCT	
ccmFc-1-R	AGTAGTCGTGACCAACAGCCA	
ccmFc-2-F	TGTTGGTCACGACTACTACAAAAAA	
ccmFc-2-R	TCAATTCCAATGCAACTTAT	
ccmFn-1-F	ATGTCTATAAATGAATTTTC	
ccmFn-1-R	AACCACGGGAGCGCCCAGCG	
ccmFn-2-F	TTGACGGAGCTCTTGCCATT	
ccmFn-2-R	CGAGCTTCTTATATGGGATC	
ccmFn-3-F	CAGGACCAGGAACCAATTCG	
ccmFn-3-R	CTACGGACGGGACGAAATCC	
cob-1-F	ATGACTATAAGGAACCAACG	
cob-1-R	AGCCAGATGAAGAAGACTGG	
cob-2-F	CATTGGGTGTACATTCTGAG	
cob-2-R	CTAAGAGACTGATCCGGTGC	
*cox*1-F	AATGCTCTGAGCAGTTTCGG	
*cox*1-R	CTAGCTTTTTGTCTCTTTGA	
cox2-F	GTATAGTAGTCTCATTGGCC	
cox2-R	TCTTTCAAAGTCACCGCTTC	
cox3-F	TATTACCAAGCACCCTCCAC	
cox3-R	TCATATACCTCCCCACCAAT	
nad1-1-F	AGGCCCGATCATGAGTGAAT	
nad1-1-R	GAAAATGATCTGGTTGGACG	
nad1-2-F	CGTCCAACCAGATCATTTTC	

nad1-2-R	TCATATTGGCATACTCTCCC	
nad1-3-F	GGGAGAGTATGCCAATATGA	
nad1-3-R	TTAAGGGAGCCATCGAAAGG	
nad2-1-F	CCCACTTCGATCAATTAGCC	
nad2-1-R	CGCTATATATTTGACACGGG	
nad2-2-F	GCTCTAGCCAAAACGAATCC	
nad2-2-R	CCGAACAATAATATTCCAGAG	
nad3-F	ATGGACAACATTTTTTTTGG	
nad3-R	TTACTCCCAATCCAAAGCAC	
nad4-1-F	TAGAACATTTCTGTGAATGC	
nad4-1-R	CCCATACCCCTATAATGATG	
nad4-2-F	GCCCATATGAATTTGGTGAC	
nad4-2-R	CACTAAGTTACTTACGGATG	
nad4L-F	ATGGATCTTATAAAATATTT	
nad4L-R	TTAACCTTGAATGCAATTTA	
nad5-F	GGGAGTCTCTTTGTAGGATA	
nad5-R	TAAGAAAACTGCTCACTAAC	
nad6-F	ATGATACTTTCAGTTTTGTC	
nad6-R	TTAGTAAATCGTGATTTGGT	
nad7-1-F	TGACGACTAGGAACGGGCAA	
nad7-1-R	CCACTAATCGTTGTTTCCAG	
nad7-2-F	CACAGCAAGCAAAGGATTGG	
nad7-2-R	CTATCTATCTACCTCTCCAAACACA	
nad9-F	ATGGATAACCAATCCATTTT	
nad9-R	TTATCCGTCGCTACGCTGTT	
orf25-F	ATGGGATTGAGTTCAACGGA	
orf25-R	CTTTCACTCACTGAAAAGTG	
OrfB-F	ATGCCTCAACTTGATAAATT	
OrfB-R	TTAGATTATGCTTCCTTGCC	
OrfX-F	GCCGAAAATGCATTTATCCT	
OrfX-R	CCACAAAGATAGCAAACTCG	
rpl16-F	ATGGAAAAACATCTTGTAAT	
rpl16-R	TTACGACCACTGAACAAACT	
rps11-F	ATGCCTCAGGAAAAAACAACG	
rps11-R	TCACGATCCCGGTAGAGGGA	
rpl2-F	ATCCAGGTCAAGGCGCAAAG	
rpl2-R	TTTCTAAGCTTACGTGCACC	
rpl5-F	ATGTTTCCACTCCATTTTCA	
rpl5-R	GATCGAAACGACTTTCCTGC	
rps1-F	ATGTTCTTGGTGGATGCAGG	
rps1-R	TCAAGTTCTTGTTTGATCTG	
rps2-F	TGAAAAAGACCAATCAAATCAAACT	

rps2-R	GGGTTCGTGCACAGATTTAC	
rps3-1-F	GGCACGAAAAGGAAATCCAA	
rps3-1-R	TATTAAAAAAGTATTGCATG	
rps3-2-F	CTCTTTCCTTTCTTCGGTGC	
rps3-2-R	ATTTCGTACGTTTCGGATATAGCAC	
rps4-1-F	ATGCCTGCATTAAGATTTAA	
rps4-1-R	AGTTGTGAGTAAGCGGAACC	
rps4-2-F	GCGGAAAACCGAAAAAGAGC	
rps4-2-R	TTATATGTTTTGGCCACGTC	
rps7-F	ATGGGGGACTTTGATGGTGA	
rps7-R	TTACCACCATCTGAAATGCG	
rps13-F	ATGTTATATATCTCAGGAGC	
rps13-R	TCATTTCCGAATTAGCTTGC	
rps19-F	ATGCCACGACGATCTATATG	
rps19-R	TTACTTTTTCCCCTTTCTGC	
atpA-F	CCCAGGGGATGTTTTTTATT	Chloroplast RNA editing assay
atpA-R	TGAAAAAAGCGTCCATTGTC	
ndhA-F	ATGATAATAGACAGGGTACAGG	
ndhA-R	TTATAGTGAAACAAGTTGGGAAG	
ndhB-F	ATGATCTGGCATGTAC AGAATG	
ndhB-R	CTAAAAGAGGGTATCCTGAGCA	
ndhD-F	ATTTTGGCTTCCTTATTGC	
ndhD-R	GCCTCTACCCTGTCAACG	
ndhF-F	ATATGCATGGGTAATCCCTC	
ndhF-R	AGTGGCTCCTAAGAAAAGTG	
ndhG-F	ATGGATTTACCTGGGCCAAT	
ndhG-R	TTATTGCCGAGCCATAGTAA	
rpl2-F	ACGGCGAAACATTTATACAA	
rpl2-R	TTACTTACGGCGACGAAGAATA	
ropB-F	ACTAAGCGTGCTATTCTCAA	
ropB-R	TTTATGGTCTAATTCCGAGC	
rps8-F	ATGGGCAAGGACACTATTG	
rps8-R	AACATAAGACTTCTCCCCCA	
rps14-F	ATGGCAAAAAAAAGTTTGATTC	
rps14-R	TTACCAACTGGATCTTGTTGCA	
ycf3-F	ATGCCTAGATCCCGTATAAATG	
ycf3-R	TTATTCAAATTCAAAGCGCTTC	
